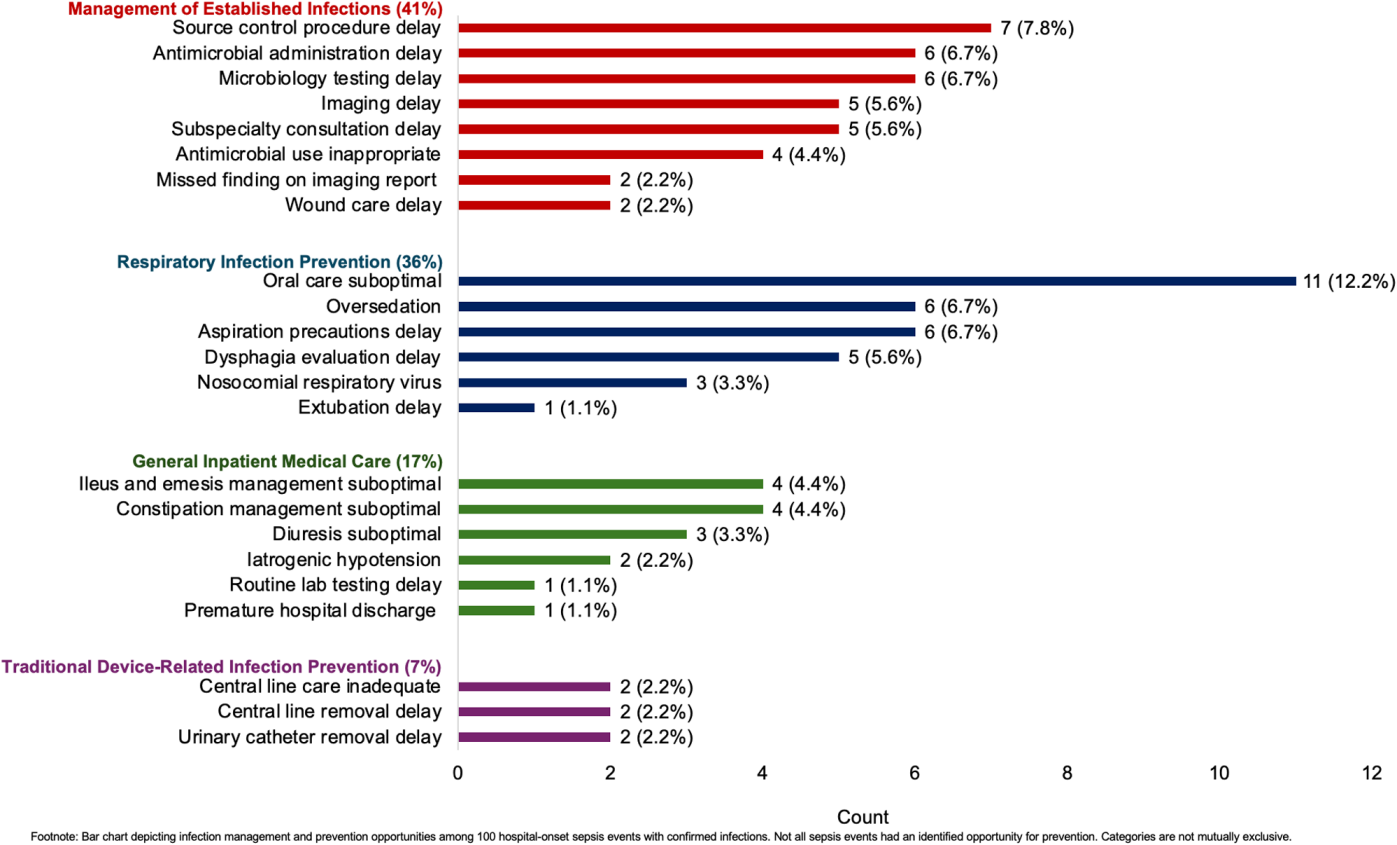# 246 Fecal microbiota transplantation results in durable long-term engraftment of donor derived anaerobic taxa and MDRO decolonization

**DOI:** 10.1017/ash.2026.10459

**Published:** 2026-06-23

**Authors:** Fizza Manzoor, Michael Klompas, Saranya Seethamaran, Christina Chan, Chanu Rhee

**Affiliations:** 1 Brigham and Women’s Hospital; 2 Harvard Medical School; 3 Harvard Pilgrim Health Care Institute; 4 Brigham and Women’s Hospital / Harvard Medical School

## Abstract

**Background:** Current US healthcare-associated infection (HAI) surveillance misses many serious nosocomial infections. Electronic identification of hospital-onset sepsis using CDC Adult Sepsis Event (ASE) criteria could expand the breadth and efficiency of HAI surveillance, but little is known about the preventability of these events. **Methods:** We identified hospital-onset sepsis events on hospital day ?4 using updated ASE criteria amongst adults admitted to 9 hospitals in Massachusetts, 2021-2023. We randomly selected 224 sepsis events to adjudicate infection probability and source (50% academic, 50% community hospitals) and then analyzed 100 confirmed infections for preventability using a 6-point Likert scale. Two physicians reviewed cases for infection source, following joint review and discussion of the first 25 cases with two senior physicians to standardize approach. All preventability assessments were classified by consensus amongst three physicians. **Results:** Amongst 224 hospital-onset sepsis events, 100 (45%) had definite or probable infection, 82 (37%) had possible infection, and 42 (19%) had no infection. Most sepsis events were due to respiratory (47%), bloodstream (16%), abdominal (13%) and urinary (9%) infections (Figure 1). Only 11 events were CMS-reportable NHSN HAIs (mostly central-line associated bloodstream infections and catheter-associated urinary tract infections). Crude inpatient mortality for hospital-onset sepsis cases was 35%. Amongst 100 cases assessed for preventability, 30% were judged potentially preventable (Figure 2), most commonly due to lapses in managing established infections (41%) including delays in source control, microbiologic testing, antimicrobial administration, imaging, and subspecialty consultation. Gaps in nosocomial respiratory infection prevention measures were also common (36%) including insufficient oral care, lack of dysphagia management, and failure to mask (Figure 3). Lapses in traditional device-related HAI prevention bundles were uncommon (7%). Some hospital-onset sepsis events may have been preventable with better general medical care (17%), including earlier recognition and management of non-infectious contributors to physiologic deterioration (e.g., earlier diuresis to optimize respiratory reserve among patients at risk for aspiration, proactive bowel management to prevent stercoral colitis and downstream infectious complications). **Conclusions:** Hospital-onset sepsis events identify serious, often fatal infections, many of which are potentially preventable. Opportunities extend beyond current HAI prevention bundles and include the need for more timely recognition and management of established infections to prevent progression to sepsis, introducing measures to prevent nosocomial respiratory infections, and improving general inpatient medical care. Incorporating hospital-onset sepsis surveillance into infection prevention and quality improvement programs could help identify actionable steps to improve the safety and quality of inpatient care.

**Figure:**
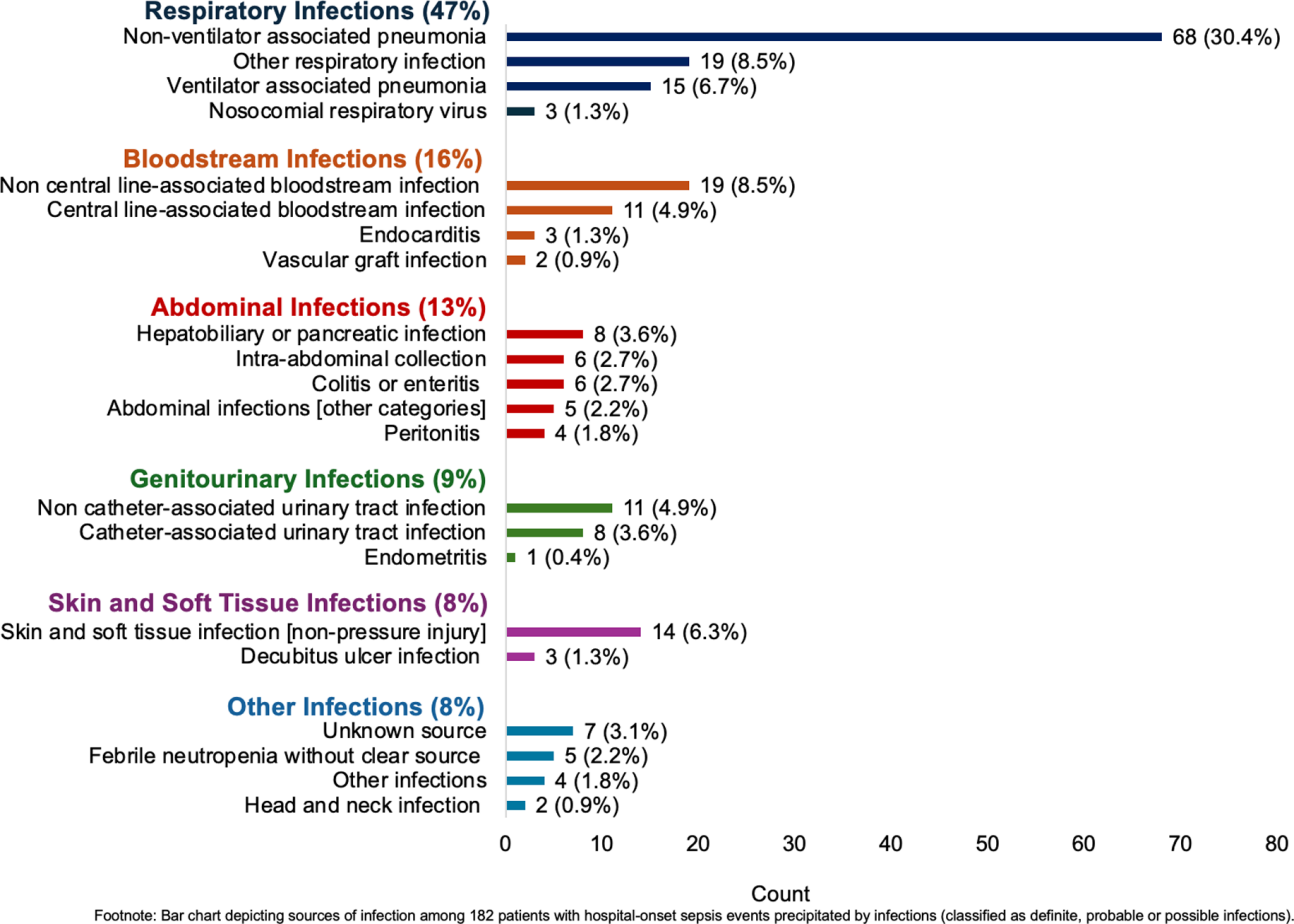

**Figure:**
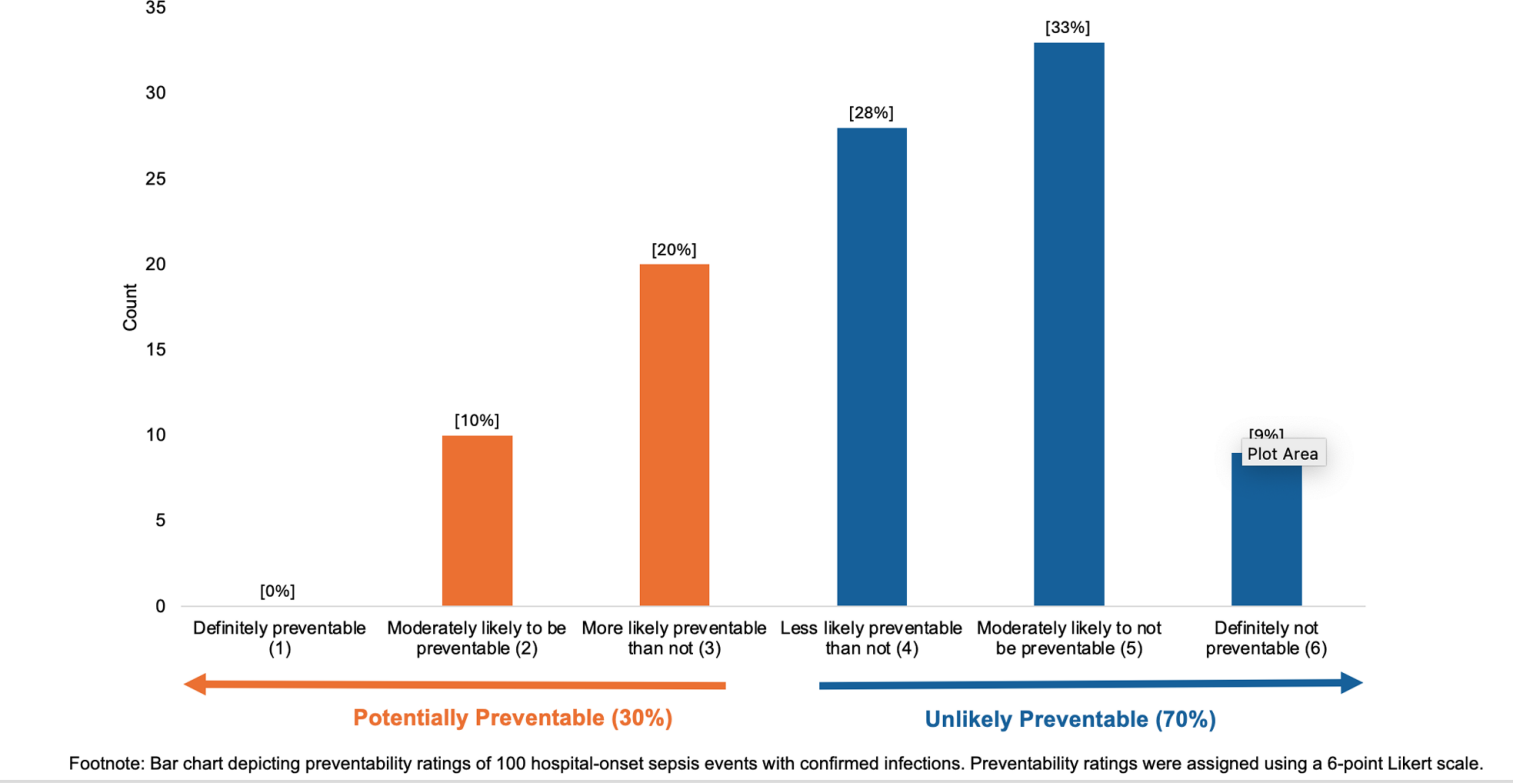

**Figure:**